# Multi-scale morphology characterization of hierarchically porous silver foam electrodes for electrochemical CO_2_ reduction

**DOI:** 10.1038/s42004-023-00847-z

**Published:** 2023-03-16

**Authors:** Hendrik Hoffmann, Melanie Cornelia Paulisch-Rinke, Marius Gernhard, Yannick Jännsch, Jana Timm, Carola Brandmeir, Steffen Lechner, Roland Marschall, Ralf Moos, Ingo Manke, Christina Roth

**Affiliations:** 1 Electrochemical Process Engineering, Universität Bayreuth, Universitätsstraße 30, 95447 Bayreuth, Germany; 2 Helmholtz-Zentrum Berlin für Materialien und Energie, Institute of Applied Materials, Hahn-Meitner-Platz 1, 14109 Berlin, Germany; 3 Department of Functional Materials, Universität Bayreuth, Universitätsstraße 30, 95447 Bayreuth, Germany; 4 Chair of Physical Chemistry III, Universität Bayreuth, Universitätsstraße 30, 95447 Bayreuth, Germany

**Keywords:** Electrocatalysis, Porous materials, Electrocatalysis, Sustainability, Electrocatalysis

## Abstract

Ag catalysts show high selectivities in the conversion of carbon dioxide to carbon monoxide during the electrochemical carbon dioxide reduction reaction (CO_2_RR). Indeed, highly catalytically active porous electrodes with increased surface area achieve faradaic conversion efficiencies close to 100%. To establish reliable structure-property relationships, the results of qualitative structural analysis need to be complemented by a more quantitative approach to assess the overall picture. In this paper, we present a combination of suitable methods to characterize foam electrodes, which were synthesised by the Dynamic Hydrogen Bubble Templation (DHBT) approach to be used for the CO_2_RR. Physicochemical and microscopic techniques in conjunction with electrochemical analyses provide insight into the structure of the carefully tailored electrodes. By elucidating the morphology, we were able to link the electrochemical deposition at higher current densities to a more homogenous and dense structure and hence, achieve a better performance in the conversion of CO_2_ to valuable products.

## Abstract (web summary)

Metal foam electrodes are promising for efficient CO_2_ reduction, but detailed characterizations of the foams at the macro and nanoscale are lacking. Here, the authors combine physicochemical and microscopic techniques in conjunction with electrochemical analyses to relate the morphologies of silver foam electrodes to their CO_2_ reduction performance.

## Introduction

According to the Intergovernmental Panel on Climate Change, global warming will not be kept below 1.5 °C, unless far-reaching reductions in carbon dioxide and other greenhouse gas emissions are being implemented in the coming decades^1^. Next to the industrially established hydrogen production by water electrolysis, further energy-conversion technologies, like the electrocatalytic nitrogen reduction reaction (N_2_RR), carbon monoxide reduction reaction (CORR) and in particular the electrochemical carbon dioxide reduction reaction (CO_2_RR) can contribute decisively to overcome this challenge^2^. Depending on the choice of the catalyst material, influencing the adsorption energies of reaction intermediates, industrially desired chemicals like syngas (CO + H_2_), formic and acetic acid, short-chain alcohols as well as hydrocarbons (methanol, ethanol, n-propanol, ethylene) can be synthesised by CO_2_RR synthetically and, hence close the carbon cycle^3–5^. While Cu is well-known for its catalytic ability to reduce CO_2_ electrochemically to a variety of products that need to be separated after electrolysis, Ag shows high selectivities in the conversion of carbon dioxide to C_1+_ products (gaseous CO + liquid HCOOH) and achieves combined faradaic conversion efficiencies close to 100%^6,7^. Although, by optimizing cell set-ups towards high electrode area-to-electrolyte ratios^8,9^ or tailoring the morphology of highly porous foams^10^, it was possible to produce small amounts of C_2+_ products also on Ag.

Accordingly, noble metal catalysts attract significant attention and demonstrated excellent performance in electrocatalysis by high electrical conductivity, catalytic activity, stability, and their ability to adsorb reaction molecules and quickly desorb the products. Disadvantages like high costs and scarcity compared to earth-abundant non-noble metal catalysts can be compensated by optimized design and resource-efficient electrode manufacturing^11^. Three-dimensional porous metal foam electrodes fulfill these demands and offer a self-supported structure, an enlarged surface area by contiguous hierarchical pore systems, and high mass transfer coefficients^12,13^. All of these characteristics play a decisive role in electrochemical performance, especially when it comes to an efficient manufacturing of gas diffusion electrodes for industrial applications. One efficient way to produce highly porous 3D structured foam electrodes via electrochemical deposition, is the Dynamic Hydrogen Bubble Templation (DHBT) method^14–16^. Shin et al.^17^ presented this rapid manufacturing technique in 2003, by which metal foam electrodes can be produced within seconds. Applying sufficiently high overpotentials, metal ions are reduced and electrochemically deposited. Accompanied by the hydrogen evolution reaction (HER), which works as a dissolving negative template, macro-porous layers with nanoscale interconnecting foam walls are formed^3,18–20^. Vital manufacturing parameters, like deposition time and current density during DHBT synthesis, are well known to influence the morphology of the macroscopic foam structures, which were frequently studied by means of scanning electron microscopy (SEM) and 2D image analysis^3,19,21–25^. Work by Zhang et al.^26^ combined laser confocal scanning microscopy (LCSM) and SEM to study effects of hydrogen bubble coalescence on the morphological behaviour of DHBT formed Cu foams. They showed the influence of the bubble-template diameter on pore size distribution and the pore density. Rudnev et al.^27^ obtained additional insight in pore depth by white light interferometry measurements and thereby also approximated foam thicknesses. Arévalo-Cid et al.^28^ combined top-down and cross-sectional SEM analysis, LCSM measurements and python-supported analysis of optical microscopy images to evaluate the effect of deposition time on the macroscopic structure of DHBT synthesised Co foams.

Yet, a combination of physicochemical and electrochemical methods to evaluate the complex structure of DHBT synthesised foams on the macro and nanoscale is still missing. To fully evaluate the complex structure of DHBT synthesised foams on the different length scales attempting for a semi-quantitative description, this work aims to shine light on porous, 3D Ag model electrodes. We provide an efficient selection of methods to analyse the morphology of these hierarchically porous electrodes qualitatively and quantitively, demonstrating that methods used individually are not enough to elucidate the complete picture. By using SEM, the growth behaviour and pore distribution of surface pores were investigated. Using the LCSM technique, 3D information was successfully converted into a calculated macro porosity of the foam electrodes. Adding morphology analysis by Focused Ion Beam tomography (FIB), 2D and 3D information to analyse Ag particle area fractions and pore size distribution of the nano-porous foam walls was obtained. Ultimately, a structure-performance relationship between the applied current density for foam deposition, the effect on the foam morphology and the catalytical CO_2_RR performance was aimed for.

## Results and discussion

### Electrochemical performance

**Fig. 1:**
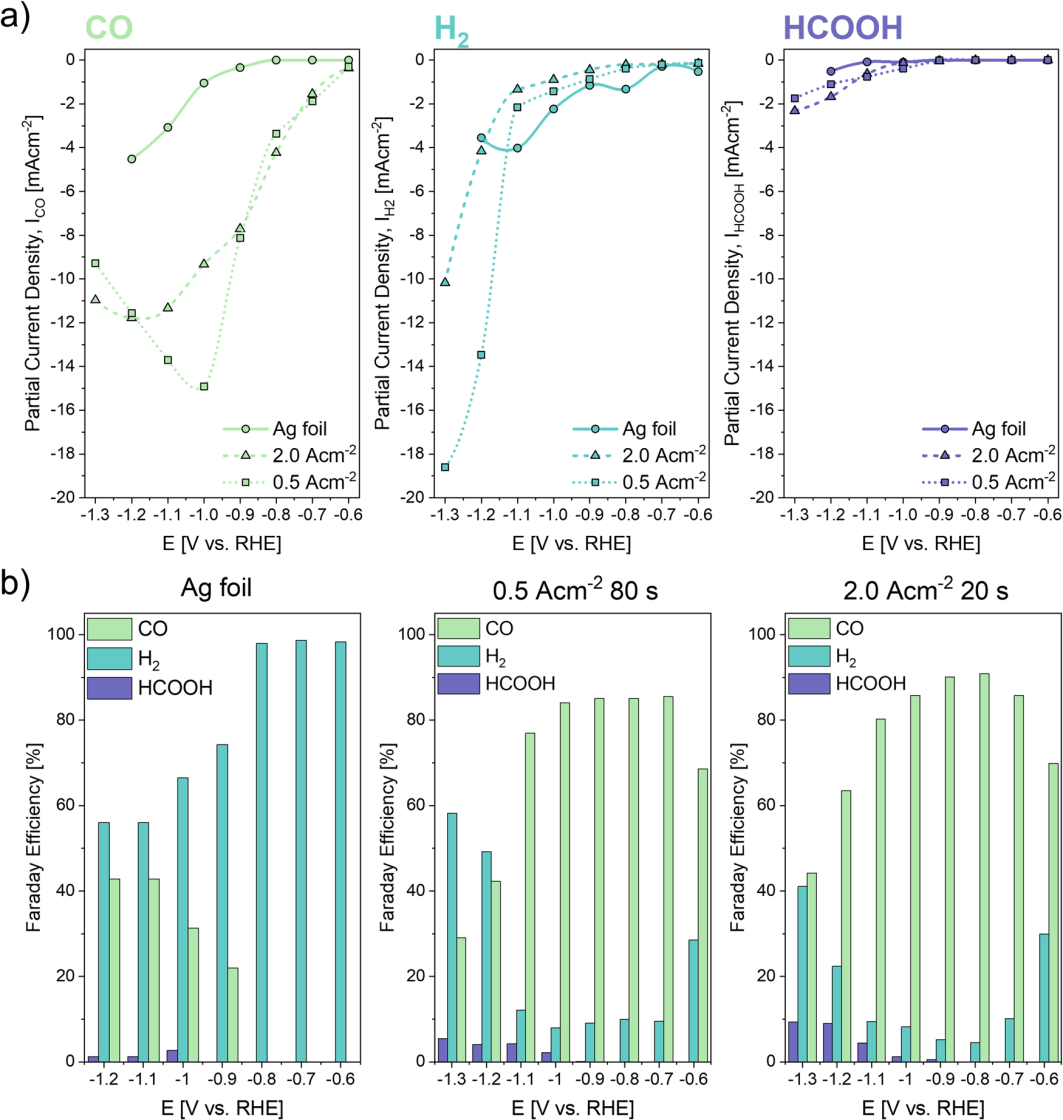
Electrochemical performance in CO_2_RR of the pristine and polished Ag foil compared to Ag foam electrodes (deposited at 2.0 Acm^−2^ 20 s and 0.5 Acm^−2^ 80 s). **a** Partial current densities and **b** faradaic efficiencies of the main evaluated products.

Partial current densities and faradaic efficiencies during CO_2_RR of the Ag foil used as substrate for the electrochemical deposition were compared to the Ag foam electrodes synthesised by the DHBT method. Evaluating their catalytic activity and faradaic efficiency (Fig. 1), carbon monoxide (CO), hydrogen (H_2_) and formic acid (HCOOH) were the main products detected with substantial amounts by GC and HPLC after 1 h of experiments. After 1 h of CO_2_RR also negligible traces of methane were detected at elevated overpotentials (Fig. S1). These results are in line with literature and were already reported in earlier studies^6,9,14^.

In Fig. 1a, the partial current densities (PCD) of the main resulting products are shown and compared. We can report, that both the I_CO_ and I_HCOOH_ of the pristine Ag foil fall below the values for the foam electrodes at every tested potential, whereas I_H2_ of the Ag foil substrate exceeds the values of the foam electrodes except for very negative potentials >−1.2 V vs. RHE. The curves of the partial current densities and the faradaic efficiencies are comparable to the work of other groups and show the same trends^10,29^. It is important to note that CO_2_RR experiments performed with Ag foils in the here used H-cell set-up could be realized only up to −1.2 V vs. RHE, because of current overload of the used potentiostat, indicating the insufficient current flow between the foil electrode and the CE mesh, due to severe gas evolution (total amount of H_2_ + CO) and high resistance due to large distance between WE and CE. Kuhl et al.^8^ and Hatsukade et al.^9^ show how optimized cell designs can have beneficial impact on the CO_2_RR performance of the tested electrodes. The CO selectivity of the pristine Ag foil was enhanced by using a sophisticated cell set-up, where CO_2_ mass transport limitations could be reduced due to an optimized CO_2_ flow through the cell and an electrode surface-to-electrolyte ratio, which allowed for the detection of small concentrations of liquid products after CO_2_RR.

At the highest tested overpotential, hcd foams reach a maximum total current density of −23.5 mAcm^−2^, which stands in line with DHBT synthesised Ag foams by Dutta et al.^10^, who used similar electrodeposition and CO_2_RR testing conditions. Lcd foams reach a maximum total current density of −29.6 mAcm^−2^, indicating an enhanced electrode performance by increased catalytic surface area. CO partial current densities (I_CO_) of Ag foam electrodes increased between starting potential and −1.2 for the hcd and −1.0 V vs. RHE for the lcd foams, respectively, whereby I_CO, max_ of lcd foams electrodes is reached at −1.0 V vs. RHE, outperforming the hcd electrodes by 200 mV. After reaching −1.0 V vs. RHE, I_H2_ of the tested foam electrodes increased to a maximum of −10 mAcm^−2^ and −17 mAcm^–2^. I_HCOOH, max_ of the hcd foam electrode does not exceed −2.4 mAcm^−2^, which here represents the maximum current density to produce HCOOH.

The FEs of Ag foil, hcd foam and lcd foam electrodes are shown in Fig. 1b. Although, the HER is predominant at each tested potential, the Ag foil shows a FE_CO_ of 21% at an onset potential of −0.9 V vs. RHE. The FE_CO_ of the Ag foil increased with elevated negative potentials but does not exceed 42.8%, which was slightly higher to what was reported from Sun et al.^29^. Compared to Ag foil electrodes, foam electrodes show FE_CO_ of 70% and 68% at −0.6 V vs. RHE, respectively, decreasing the CO-onset potential of the Ag substrate electrodes by 300 mV. Although the FE_CO_ stay on a high level (>80%) within a potential window of 400 mV for hcd foams and 300 mV for lcd foam electrodes, we see a fast decrease in FE_CO_ for applied potentials below −1.1 V vs. RHE. For hcd foams, the CO_2_RR remains favoured, and it outperforms the competing HER at each tested potential compared to lcd foams, where HER prevails starting at −1.2 V vs. RHE. Foam electrodes synthesised with even higher current densities by Dutta et al. keep high FE_CO_, at elevated potentials up to −1.3 V vs. RHE^10^. For all tested electrodes, the production of HCOOH can be reported, with increasing FE_HCOOH_ at more elevated overpotentials, whereas the FE_CO, max_ is reached by hcd foam electrodes at −1.3 V vs. RHE. FE_CO_ as a function of the applied current density during potentiostatic operations shows that hcd foam electrodes produce CO more efficiently by operation up to current densities of 10.36 mAcm^−2^. Although FE_CO_ for lcd foams is slightly higher above that value, both foam electrodes experience a sharp decrease in FE_CO_, where CO_2_RR is fading (Fig. S10). As the reported data show distinct differences in the electrochemical CO_2_RR performance and selectivity between pristine 2D Ag foil and electrochemically synthesised foam electrodes, but also between the foam electrode structures themselves, the morphology of the tested electrodes needs to be investigated closely to arrive at a decent structure-property correlation. Overall, the hcd foam electrodes seem to perform better with respect to CO_2_RR.

### Dynamic hydrogen bubble templation

**Fig. 2:**
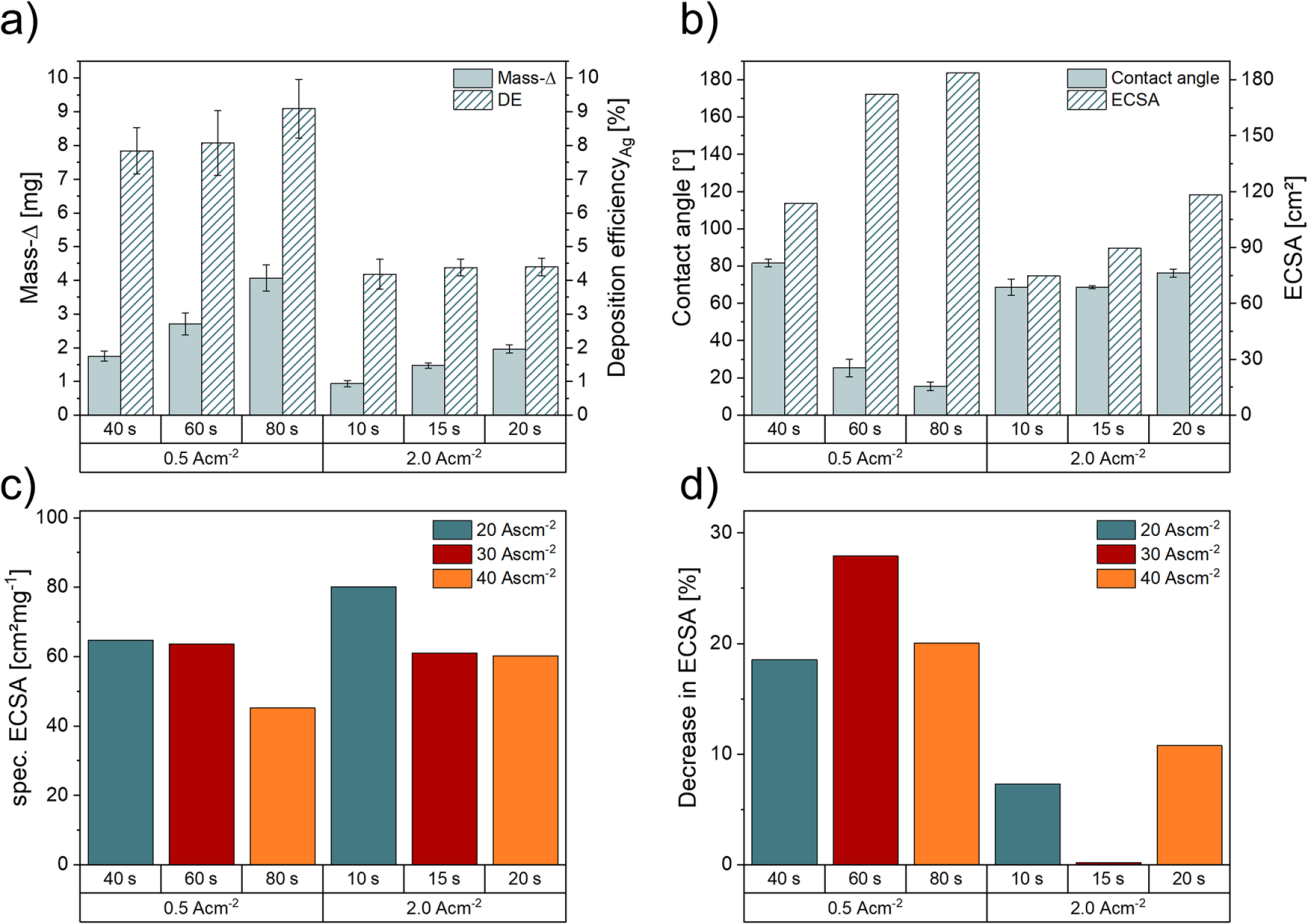
Influence of the applied DHBT parameters on the deposited silver mass, deposition efficiency, surface contact angle and resulting electrochemical surface area. Mass-increase and Ag deposition efficiency of Dynamic Hydrogen Bubble Templation method (**a**), contact angle and absolute values of the electrochemical surface area (ECSA) of all tested samples (**b**), the resulting specific ECSA (**c**) and the decrease in ECSA after 1 h of CO_2_RR at each tested potential (**d**). Error bars correspond to the average of at least two individual measurements on two different samples.

The galvanostatically deposited mass increases for hcd foam and lcd foam electrodes with increasing deposition time and electric charge densities, showing the highest mass differences at 20 s and 80 s, respectively (Fig. 2a). Comparing these results regarding electrical charge densities, it becomes obvious that galvanic deposition at low current densities shows higher mass difference values compared to the hcd foam samples. This result can be supported by the calculated FEs of the DHBT synthesis. Lcd foams show a more efficient Ag deposition (*η* = 8–9%) compared to hcd foam electrodes (*η* = 1.9–2%), which can be explained by enhanced HER at elevated current densities. Nikolić et al. already reported the effect of increasing overpotentials on the H_2_ efficiency and the increase in current density during metal electrodeposition^30,31^. In Fig. 2b, the influence of the DHBT parameters on the resulting contact angles and calculated ECSA values is demonstrated. Foam electrodes that were deposited at high current densities, show slowly decreasing hydrophilicity with increasing deposition time. The foam electrodes synthesised at low current densities show the most hydrophilic values at 40 s and less hydrophilicity at deposition times above 60 s. The ECSA increases with increasing deposition time for both galvanostatic parameters, with foam electrodes deposited at low current densities having higher values than foam electrodes deposited at higher current densities, comparing electrodes with the same applied charge densities during synthesis. Combining the results of mass deposition and the absolute ECSA values, hcd foams show a higher specific ECSA than lcd foams comparing the same charge density, except for intermediate deposition times (60 s). Here, hcd and lcd show almost the same specific ECSA values. (Fig. 2c). Analysis before and after CO_2_RR reveals a more stable behaviour of hcd foam electrodes compared to lcd foams, as they show a significantly lower performance decrease after 1 h of CO_2_RR operation (Fig. 2d).

In addition to morphological characteristics, which are at the center of our investigations, the analysis of the crystal structure of the electrodes is required to exclude differences in the tested samples.

### Crystal structure analysis

**Fig. 3:**
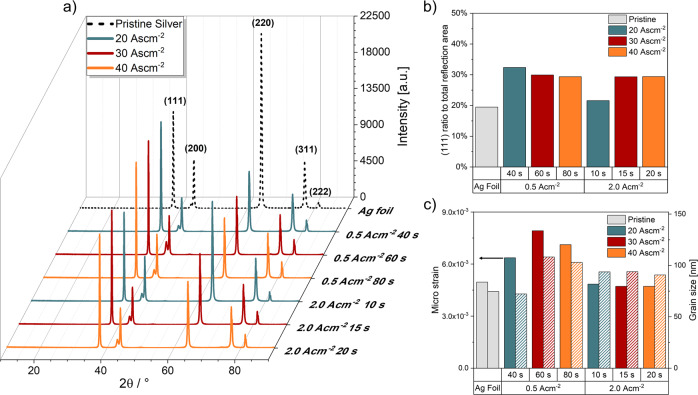
X-Ray diffractogram of the reflex intensities of all tested samples compared to the mechanically polished pristine Ag foil. **a** Normalized intensities of the reflexes vs. the (111) reflex (**b**) and micro strain and Ag grain size of the Ag crystallites calculated by the Williamson-Hall method and Scherrer-equation, respectively (**c**).

Diffractograms showing the (111), (200), (220), (311) and (222) reflections of Ag (ICDD: 04-001-2617), were taken for all samples (Fig. 3a). At 43.3° 2θ, a reflection with varying normalized intensities was observed throughout all samples and was identified as the (110) reflection of austenite (ICDD: 04-002-3692) from the sample holder steel disc. It has been argued that Ag may deposit preferably in (111) and (200) orientation, since these orientations show the lowest surface energy. However, the (311) orientation is theoretically even more preferred when regarding the combination of surface and strain energy minimization^32,33^. In our study, we found that the (111) orientation is preferred for the Ag deposition under DHBT conditions. Figure 3b shows that the proportion of the (111) reflection to the sum of all reflections increases significantly from 19.5% for the pristine Ag foil to 29.4% for the Ag foams deposited with a charge density of 40 Ascm^−2^, which is a 51% increase. The micro strain of the deposited foams was investigated by the Williamson Hall method. We found that a micro strain of ~5 × 10^−3^ is already present in the pristine foil which may originate from the foil rolling during manufacturing. The micro strain increases up to 8 × 10^−3^ for the lcd foam at 60 s deposition time. However, the micro strain shows a decreasing trend for all hcd foams as shown in Fig. 3c. If larger grains incorporate more defects resulting in an increased micro strain, we expect that at 0.5 Acm^−2^ grain sizes should be larger. Grain sizes calculated from the (111) (reflection in Fig. 3c) show indeed that the grain sizes of lcd foams at 60 s (108 nm) and 80 s (103 nm) are slightly larger than those of the hcd foams at 15 s (94 nm) and 20 s (91 nm). Overall, deposited Ag structures under DHBT conditions show a similar nanostructure to Ag-deposited thin films. From this observation, we can conclude that the high overpotential during deposition does not introduce increased micro strain into the foam structures.

Although X-Ray diffractograms can be used to characterize nanostructures of DHBT-deposited Ag, neither qualitative nor quantitative characterization of the porous micro- and macrostructures are possible. The X-Ray diffractograms and thereof derived crystallite sizes correlate with the parameters used in depositing the Ag structures, but overall, mainly corroborate similar crystal structures and growth behaviours amongst the different foam structures. Thus, observed performance differences do most probably not originate from various crystalline features or preferred orientation and strain effects.

### Specific surface area

To derive a more quantitative value of the surface area of porous foam materials, the BET method is a routinely applied procedure. Results are shown in Table 1. The lcd and hcd foams show the largest specific surface area at low deposition times. With increasing deposition time, a decreasing specific surface area can be seen for lcd and hcd foams, although hcd foams do show a slight increase after 20 s deposition time. The data fitting is very difficult and the values for the surface are very low, therefore the interpretation of differences of the electrodes synthesised at high current densities may overestimate the real values. The comparatively low specific surface is not a result of low porosity in the foams, rather the high density of 10.5 gcm^−1^ of Ag^34^ is responsible for the low specific surface areas.

**Table 1:** Specific surface areas of the porous foam electrodes calculated by BET method.

Parameter	Sample					
i [Acm^−2^]	0.5			2.0		
t [s]	40	60	80	10	15	20
σ [Ascm^−2^]	20	30	40	20	30	40
BET_foam_ [m^2^g^−1^]	3.4	1.8	1.3	0.4	0.2	0.3

The differences and the decreasing trend of the specific surface areas of lcd and hcd foam samples can be explained by the fact that only very low masses of Ag were deposited. This resulted in very small absolute surface areas (0.003 – 0.005 m^2^), which are at least one order of magnitude below assessable surface areas by Krypton physisorption^35,36^. Therefore, we make use of other methods to arrive at meaningful conclusions and support our measurements with better significance.

### 2D morphology study

**Fig. 4:**
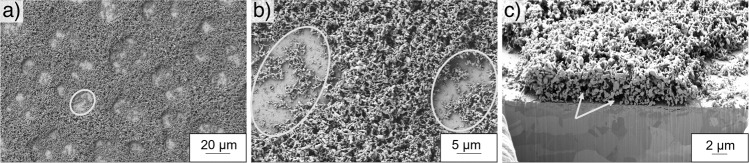
SEM and FIB-SEM images taken from a porous foam model electrode (electrochemically deposited with 2.0 Acm^−2^ for 5 s). The images show an overview of the macro-porous region (**a**) and the transition from macro-porous craters to nano-porous foam walls (**b**) and (**c**).

SEM and FIB-SEM images of the model electrodes porous structures are shown in Fig. 4. Qualitative analysis of the foam morphology shows the coexistence of two porous regions and can be subdivided in macro craters formed around agglomerated hydrogen bubble templates and nano-porous walls, that connect the observed macro craters.

**Fig. 5:**
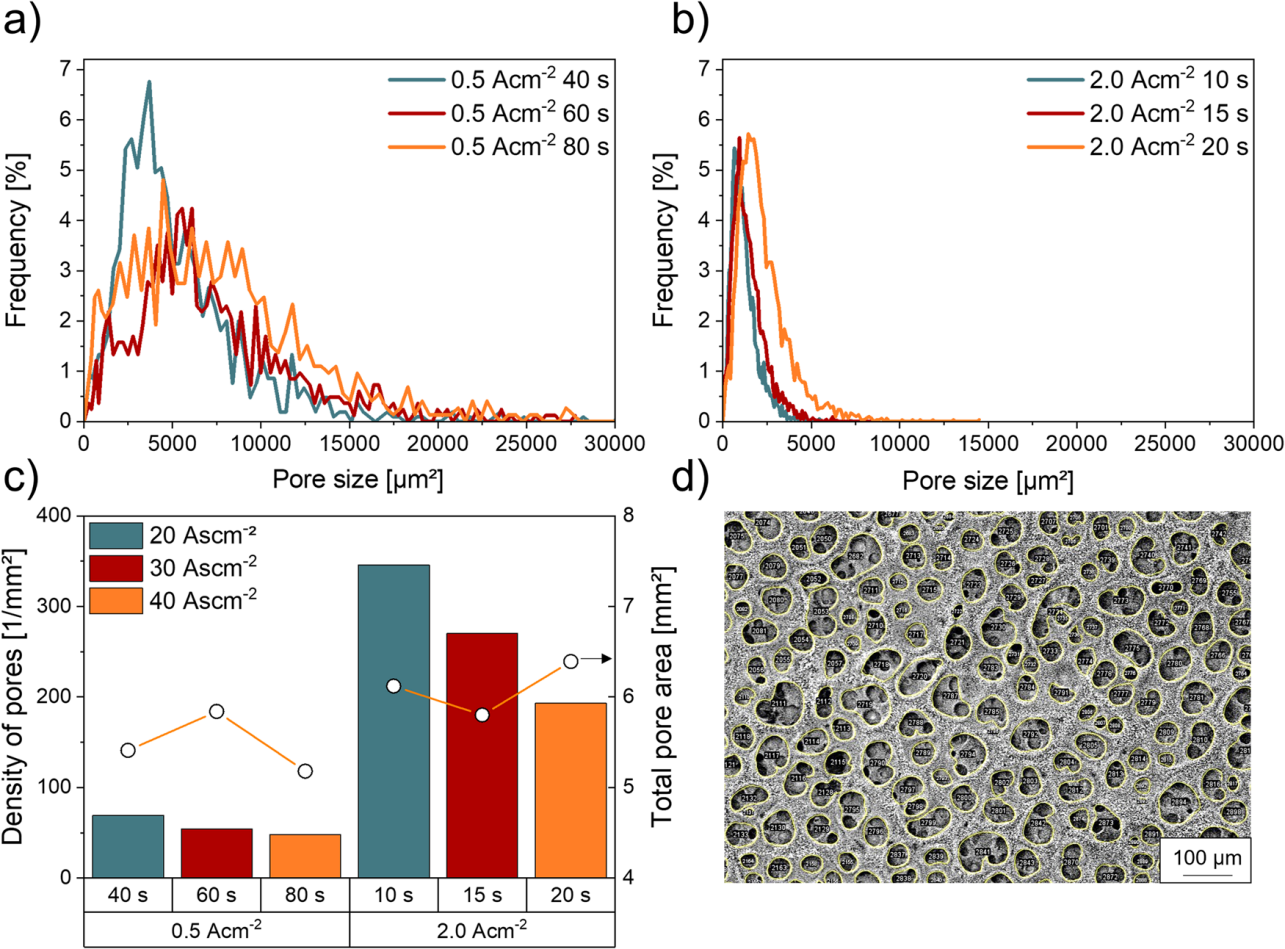
Influence of the applied DHBT parameters on the pore size distribution, pore density and the size of the generated surface pores. **a** Pore size distribution of the tested samples with 2.0 Acm^−2^ and **b** with 0.5 Acm^−2^ at increasing deposition times, **c** pore density and total pore area of all tested samples, **d** one of 16 exemplary SEM images stitched together to a mosaic, taken for analysis.

To quantify the macro crater size, which is accessible from the surface view, 16 SEM mosaic images were taken and stitched together, resulting in an analysed area of 15.29 mm² for each sample (Figs. S2–S7). The contours of the surface pore craters of each sample were manually traced and results are presented in Fig. 5 showing that the pore sizes of the surface macro craters of the foam electrodes synthesised at high current densities are ~50% smaller compared to the foams electrodeposited at low current densities. As can be seen in Fig. 5a, the maximum of the relative frequency of the pore distribution of the hcd foams shifts from 653 µm^2^ to 947 µm^2^ when deposition times are increased from 10 s to 15 s and jumps up to 1447 µm^2^ when deposition times of 20 s are applied. The maximum of the relative frequency of lcd foams immediately broadens and shifts from 3693 µm^2^ to 5547 µm^2^ after deposition times of 60 s and stays almost constant with a slight decline to 4470 µm^2^ after 80 s (Fig. 4b). As pore distributions broaden at both parameter sets with increasing deposition times, this result underlines the fact that hydrogen bubbles, that are used as negative templates to form the porous craters, are agglomerating over time before being released and detaching from the surface. This result is supported by the declining pore densities at increasing deposition times (Fig. 5c).

It should be noted that for the same charge densities, the pore densities, created at high deposition current densities are much higher than the pore densities created at lower deposition current densities. Comparing these results with the correlating pore areas, significant differences in the growth behaviour can be derived. While lcd foams show a decreasing pore density and increasing total pore area between 40 s and 60 s deposition time, a surface pore coalescence is suggested. Between 60 s and 80 s, the parallel decrease in pore density and total pore area is a result of the growing proportion of interconnecting nanoporous- foam walls. Analysis of the samples deposited at high current densities shows a completely opposite behaviour, where nanoporous walls form in the first step, followed bubble coalescence after 15 s (Fig. 5c). With the SEM method, only surface information could be generated, but information about the depth of the pores remains off scope. Therefore, the 2D information needs to be supplemented by an additional vertical analysis.

### 3D morphology study

**Fig. 6:**
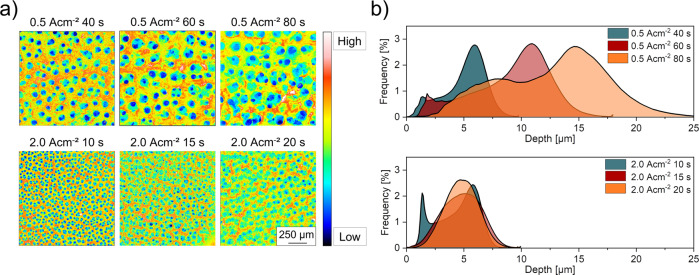
Laser confocal scanning microscopy (LCSM) images of all tested samples and resulting pore-depth distribution. **a** Coloured 2D LCSM images of low current density and high current density foam electrodes with high (red to white) and low (blue to black) pore depths, **b** distribution of the pore depths in relation to the applied charge densities of all tested samples.

We used the LCSM method to analyse the macro craters of the foam electrodes. Figure 6 shows the results of the LCSM measurements performed on lcd foam samples deposited for 40 s, 60 s and 80 s and on hcd foam samples for 10 s, 15 s and 20 s, respectively. It is shown that the depth range in which pores are formed when deposition is applied at low current densities reaches up to 25 µm, whereas the depth range of foam electrodes deposited with high current densities remains constant (~ max. 10 µm). Foam deposition at low current densities taking place at 60 s and 80 s increases the thickness of the lcd foams and the depth of pores accordingly. The pore depth distribution of the lcd foams remains bimodal, independent of the deposition time. Looking at foam electrodes deposited at high current densities, this is not the case. The bimodal distribution is turning into a monomodal distribution after 15 s and 20 s, respectively. Comparing the charge densities, applying 20 Ascm^−2^ for a duration of 10 s show higher frequencies of flat pores (~1.5 µm) and less deep pores than for deposition times of 40 s. An explanation for this behaviour could be a different foam growth mechanism on the macro-scale. While lcd foams grow predominantly in a vertical direction to the substrate and their pore distribution remains bimodular, hcd foams tend to close flat macropores by forming parts of nano-porous foam walls when the deposition time is extended. Additionally, deep pores grow together as seen in the SEM images and form a less deep but monomodal distribution.

**Fig. 7:**
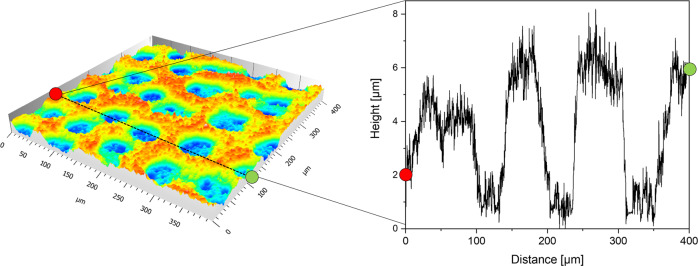
Partial 3D reconstruction of a foam electrode and the profilometric information gained by a horizontal line scan along the *x*-axis. The information of an area of 400 µm × 400 µm, obtained by laser confocal scanning microscopy was taken to calculate the macro porosity of all tested electrodes.

In Fig. 7a partial 3D reconstruction (400 µm × 400 µm) of an Ag foam electrode and a horizontal line scan along the measured *x*-axis are shown. As demonstrated by the profilometric information given by the line scan, it was possible to get the overall 3D coordinates of the foam electrodes. 3D reconstructions of every tested foam electrode are shown in Fig. S9. By Eqs. (8) and (9) the volume of a bulk Ag cuboid and the volume of the macro-porous Ag foam, which represents the volume under the curve, was calculated. The ratio of these values was given by Eq. (10) and represents the calculated macro porosity of the tested samples (Table 2).

**Table 2:** Calculated porosity of the foam electrodes by laser confocal scanning microscopy data of all foam electrodes.

Parameter	Sample					
i [Acm^−2^]	0.5			2.0		
t [s]	40	60	80	10	15	20
σ [Ascm^−2^]	20	30	40	20	30	40
V_total_ [mm^3^]	0.011	0.019	0.031	0.010	0.012	0.011
V_Ag_ [mm^3^]	0.006	0.010	0.015	0.005	0.006	0.006
Φ [%]	56.3	52.3	49.4	48.6	52.0	51.5

The calculated porosity of the lcd foam electrodes shows the highest values at 40 s deposition time. Increasing deposition times of 60 s and 80 s led to a decreasing calculated porosity, reaching the minimum macro porosity of 49.4% for lcd foams. The foam electrodes deposited at higher current densities show the opposite trend, although the calculated porosity after 15 s and 20 s is very similar (Table 2).

While the combination of SEM and LCSM techniques gave insight into the macro porosity of the foam electrodes, the nano-porous foam walls still need to be quantitively described. Therefore, all samples were analysed by FIB tomography.

### Morphology of nano-porous walls

**Fig. 8:**
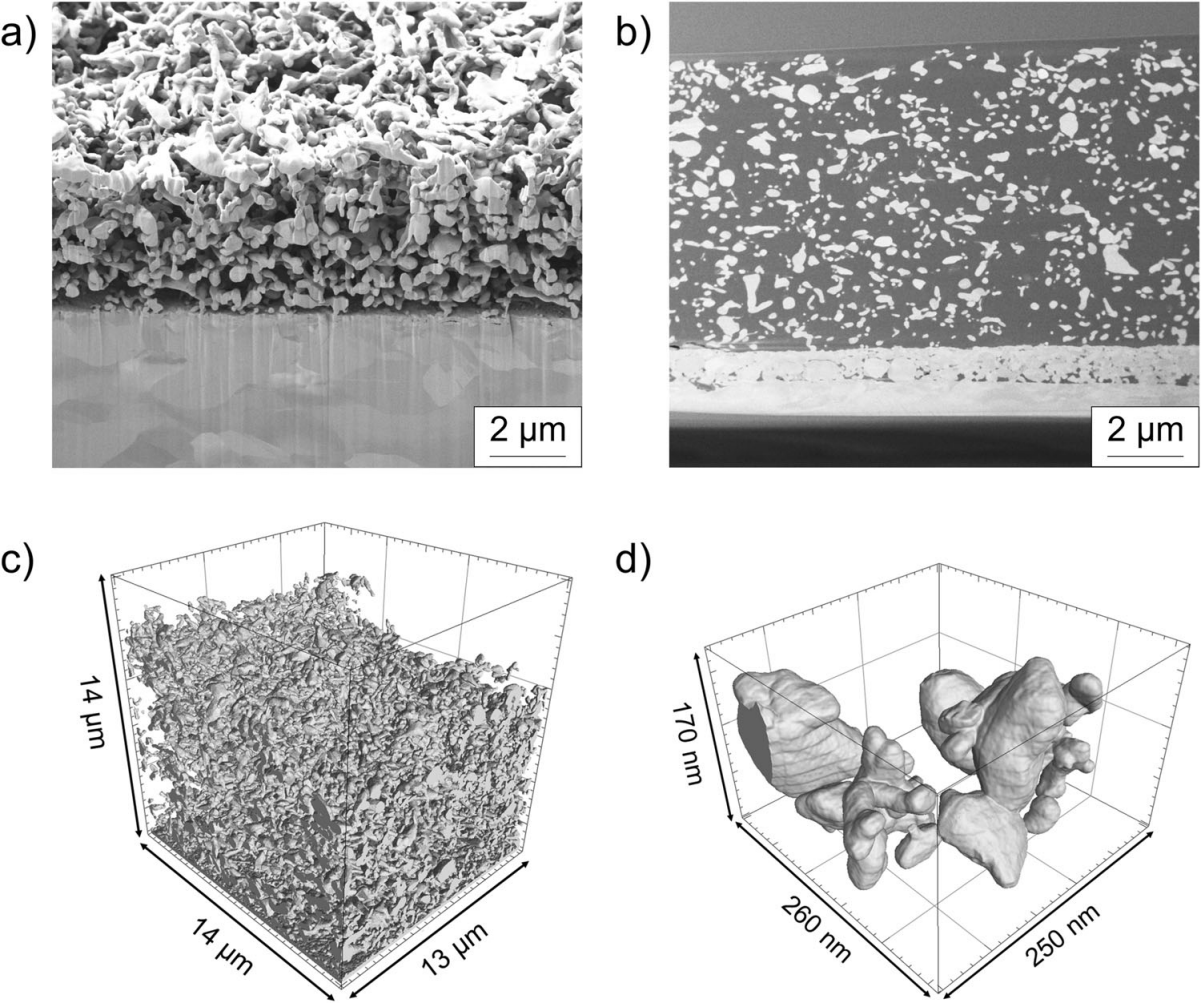
Microstructure of nano-porous foam walls. **a** FIB-SEM, Ag foam, deposited with 0.25 Acm^−2^ for 40 s, not embedded in resin, **b** FIB-SEM, Ag foam, deposited with 0.25 Acm^−2^ for 40 s embedded in resin, **c** 3D reconstruction of a foam deposited with 0.5 Acm^−2^ for 80 s, **d** enlargement of **c**, single particles of the foam.

To get detailed information about the nanostructure of the Ag foams, the samples have been measured by FIB cutting and tomographies. Figure 8a shows the cross section of a nano-porous foam wall, with the Ag substrate foil in the lower part and the deposited Ag particles on top. Initial attempts to measure the non-stabilized foams (by means of FIB tomography) failed, as particles flew off the cutting surface, or bent away. Therefore, the Ag foams were embedded in resin to improve their stability during the cutting process (Fig. 8b).

**Fig. 9:**
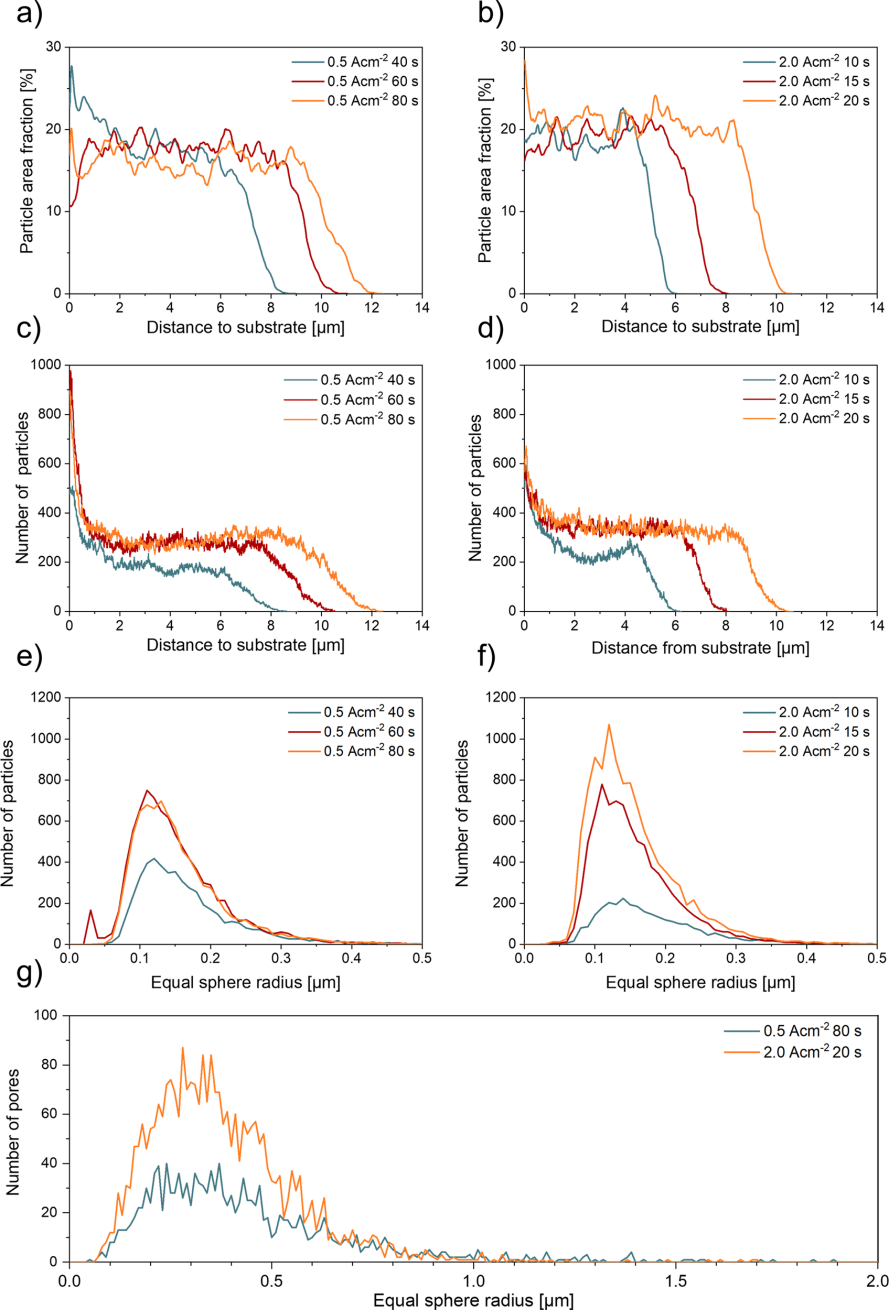
Morphological characteristics of the nano-porous foam walls. Graphs **a** and **b** show the Ag particle area fraction in dependence on their distance to the Ag foil substrate deposited with 0.5 Acm^−2^ and 2.0 Acm^−2^, **c **and** d** number of particles in dependence on the substrate distance of foams deposited with 0.5 Acm^−2^ and 2.0 Acm^−2^, **e** and **f** equal sphere radius of foam particles deposited with 0.5 Acm^−2^ and 2.0 Acm^−2^, **g** equal sphere radius of pores of the foams deposited with 0.5 Acm^−2^ and 2.0 Acm^−2^.

The FIB tomographies were reconstructed, segmented, and subsequently used to quantify the morphology and distribution of the Ag particles and the pore sizes inside the nano-porous foam walls (Fig. 9). Figure 9a, b show the Ag particle area fraction in the analysed volume as a function of their distance to the foil substrate surface. In the first few nm close to the surface, only lcd foams deposited for 40 s show a higher particle area fraction compared to hcd, synthesised with the same charge density. Foam electrodes, deposited with 30 and 40 Ascm^-2^ show higher Ag particle area fractions, when deposited at higher current densities. Across the foam thickness the particle area fraction fluctuates between 15 and 20% for lcd foams and between 17 and 22% for hcd foams. Close to the foam surface the particle area fraction is decreasing rapidly for all samples. These characteristic features are similar for all deposition times; however, it is visible that the foam thickness is increasing with longer deposition times. With regard to the applied deposition current density, slightly higher particle area fractions are observed for the hcd foam electrodes, with the highest values obtained at 2.0 Acm^−2^ for 20 s.

Figure 9c, d show the number of particles in the analysed volume in dependence of their distance to the foil substrate surface. To ensure the comparability of the results, a normalized geometric base area for the tested foams was analysed. For each foam, an area of 14 × 10 µm² was applied, however, the third dimension of the analysed volume, the foam thickness, was not normalized, as it is a result of the deposition process itself. Lcd foams, show a significantly higher particle density in the first 1 µm near to the substrate (Fig. 9c). Across the foam thickness, the number of particles remains roughly the same for each foam, until the last ~2 µm near the foam surface, where the number of particles decreases strongly. The results also show less particles for the foam, which was produced with a deposition time of 40 s, than for the foams with deposition times of 60 s and 80 s. Hcd foam electrodes show the same characteristics (Fig. 9d): A higher number of particles is visible in the first 1 µm near the substrate along the entire foam thickness, where the particle numbers stay at the same level until the last 2 µm near the foam surface are reached, where the particle number decreases rapidly. Concentrating on the particle loading at the same distance to the substrate, the particle loading increases with increasing deposition times for both deposition current densities, whereas hcd foams deposited with the same charge density show a higher particle loading. These results demonstrate that the Ag foams do not only grow in thickness, but particles grow within the nano-porous foam walls with increasing deposition time, indicating a more compact and dense nano foam wall structure by high current density deposition.

To analyse the particle volume of the different foams, a three-dimensional distance transformation followed by a watershed transformation was applied to separate overlapping particles. Afterwards, the particle volume has been measured and the equal sphere radii were calculated. Figure 9e, f show histograms of the equal sphere radii distribution. To ensure the comparability between the different foams, the calculation was carried out for volumes with uniform base area of 14 × 10 µm². The third volume dimension is determined by the individual foam thickness. The histograms for the results of the lcd foams show each a maximum at equal sphere radii between 0.11 and 0.13 µm, independent of the deposition time. However, the height of the maximum is smaller for the foam deposited for 40 s than for the other foams, which is a result of the smaller foam thickness, thus, a smaller number of particles are in the analysed volume. Although the lcd foams show the same trend compared to the hcd foams, the heights of the two maxima of the foams, which were deposited for 60 s and 80 s, are very close. Most likely, the same height of the maxima is due to a more heterogeneous morphology compared to the hcd foams. For foams deposited at 2.0 Acm^−2^ the histograms show maxima for the equal sphere radii between 0.12 and 0.14 µm independent of the deposition time. The height of the maxima is decreasing with decreasing deposition times, caused by the decreasing particle number in the analysed volume, which is attributed to the decreasing foam thickness. Comparing the foams produced at 0.5 Acm^−2^ and 2.0 Acm^−2^, the equal sphere radius of hcd foams is slightly higher.

The pore size distribution was analysed for the foams produced at 0.5 Acm^−2^ for 80 s and 2.0 Acm^−2^ for 20 s. These conditions with the longest deposition time for each series were chosen for their high foam thicknesses and, thus, a possibly high analysis volume. The pores were separated by calculating a three-dimensional distance transformation followed by a watershed analysis. To ensure the comparability between both foams, the pore size measurement and the calculation of the equal sphere radii were carried out for volumes with a uniform base area of 14 × 10 µm² (Fig. 9g). The foam deposited at 0.5 Acm^−2^ shows a broad pore size distribution with a maximum between 0.22 and 0.37 µm. The foam produced at 2.0 Acm^−2^ shows a smaller distribution of pore sizes with a maximum between 0.28 and 0.35 µm. Accordingly, it can therefore be concluded that the foam, produced at 2.0 Acm^−2^, is more homogeneous and densely structured than the foam, produced at 0.5 Acm^−2^. For a graphical illustration of the above detailed information, see Fig. S11.

### Correlation between morphology and CO_2_RR performance

We have observed that CO_2_ reduction to C_1+_ products is favored by the foams obtained by high current density electrodeposition. For instance, the partial current densities (normalized by ECSA) of these hcd foams outperformed the lcd foams at all tested potentials (Fig. S10). However, we also observed a decrease in performance over electrolysis time. Post-mortem analysis revealed that at elevated testing potentials (-1.3 V vs. RHE), both foams underwent severe changes in their nanostructures. Sharp corners and fine structured Ag needles in pre-CO_2_RR state became rounded and seemed to agglomerate after potentiostatic operation for 1 h, which most likely is the result of erosion corrosion due to severe gas formation at the surface, which ultimately reduced the number of catalytic centres (Fig. S8). This behaviour of Ag foam electrodes, synthesised by the same DHBT method, is known and had been reported elsewhere^10^.

We propose that the observed differences in electrochemical behaviour and selectivity are directly linked to the foam structure. However, only the combination of advanced and sophisticated characterization methods allowed to arrive at a decent structure-property correlation. In particular, a semi-quantitative approach as undertaken in this study is required, as microscopic analysis is often affected by artefacts and subjective conclusions. Here, semi-quantitative results obtained by FIB-SEM tomography unraveled higher values of particle area fraction, number of Ag particles in relation to their distance of the substrate and to the equal square radius of hcd foams than lcd foams. Although low and high current density deposition increased the thickness of the foams by increasing deposition time, SEM (Fig. 5), LCSM (Fig. 6) as well as FIB-SEM tomography results (Fig. 9) indicated that hcd deposition leads to foam structures with deeper pores and microstructures with higher macroporosity, consisting of a sharp macro pore distribution and a higher nano-porosity than lcd foams.

Only the combination of all these results put us in a position to arrive at decent structure-activity correlations. We conclude that a more densely structured electrode with higher, more stable specific surface area and elevated CO_2_RR activity towards C_1+_ products can be obtained by increasing deposition current densities when the DHBT method is applied (Table 3). Table 3 illustrates the differences between the two foams with respect to activity parameters.

**Table 3:** Summary of structure relationship parameters between the Dynamic Hydrogen Bubble Templation process and CO_2_RR performance.

Lcd deposition	superior	Hcd deposition
	Ag deposition efficiency	
	Specific ECSA	
	Degradation in ECSA after CO_2_RR operation	
	Density of Ag nanoparticles	
	C_1+_ product formation	

## Conclusions

A strategy to systematically analyse the morphology of highly porous 3D Ag foam model electrodes was derived and discussed. The fast and efficient DHBT deposition results in hierarchically porous foam electrodes with high surface areas and maximized electrochemically active sites. By combination of electrochemical and microscopic methods it was possible to perform a systematic screening of the nanoscale catalysts. First, the complex 3D structure of the highly catalytically active Ag foams towards CO_2_RR was elucidated by FIB-SEM analysis, evidencing a two-scale porous volume that can qualitatively be divided into macro craters and their connecting nano porous walls. Analysis of the crystal structure demonstrated that the observed performance differences are most likely not due to different crystalline features or preferential orientation and strain effects. The macro craters were analysed using the SEM and LCSM techniques, resulting in a narrower pore distribution of hcd foam electrodes compared to lcd foams. LCSM analysis enabled us to calculate a macro porosity value, which is by far more accurate than the estimated bulk porosity by volume ratios. FIB-SEM tomography was used to analyse the particle size and the pore size distributions of the interconnected nano-porous foam walls, obtained at the different stages of template-supported electrode processing. By this technique, it was possible to elucidate the growth mechanism of the foams as it correlates with its deposition parameters. The analyses of the nano-porous foam walls show that both kinds of foams exhibit homogeneous particle size distributions across the foam thicknesses, independent of the deposition times. However, near to the substrate and the foam surface significant differences in the number of particles were revealed. The analyses of the pore size distribution showed that the foams produced at 2.0 Acm^−2^ exhibit a more homogeneous structure than the foams, produced at 0.5 Acm^−2^. Hence, increasing the DHBT deposition current densities leads to more densely structured electrodes with higher, more stable specific surface area and increased CO_2_RR activity towards C_1+_ products. The results obtained by the smart combination of different characterization methods built a strong basis for a structure-performance relationship, which will aid in the knowledge-guided development of technical GDEs for industrial CO_2_RR in the future.

## Experimental section

### Preparation of porous Ag foams

Six individual Ag foam model electrodes were produced by applying the DHBT method in a three-electrode set-up. The substrates (working electrode, WE), used to produce the porous Ag electrodes (polycrystalline Ag foil, 0.1 mm thickness, 99,995%, Alfa Aesar) were mechanically polished, cleaned with acetone and ultrapure water, before they were dried in air. A platinum foil (0.1 mm thickness, 99,995%, Goodfellow) and a Ag/AgCl-electrode (Biologic) served as counter electrode (CE) and reference electrode (RE), respectively. An acidic aqueous solution served as the electrolyte (0.02 M Ag_2_SO_4_ + 1.5 M H_2_SO_4_). The DHBT synthesis took place in a glass beaker, applying galvanostatic mode using a potentiostat (Gamry Ref 3000, Gamry Instruments, USA). Two different current densities (2.0 Acm^−2^, 0.5 Acm^−2^) were used, while the deposition times were increased from 10 s to 15 s and 20 s, and from 40 s to 60 s and 80 s, respectively, to maintain the same applied charge densities of 20 Ascm^−2^, 30 Ascm^−2^ and 40 Ascm^−2^. Foams deposited with 2.0 Acm^−2^ and 0.5 Acm^−2^ will be referred to as high current density foams (hcd) and low current density foams (lcd), respectively. All model electrodes were masked by isolating Kapton tape to control deposition on a geometric area of 10 × 10 mm² (A_geo_). During deposition, a working to counter electrode distance of 8 mm was maintained. After deposition, all catalysts were removed from the electrolytic bath immediately, rinsed with ultrapure water, and dried in air.

### Electrochemical CO_2_ reduction and product analysis

The CO_2_RR experiments were performed in a three-electrode set-up at room temperature in a custom-built gas-tight two-chamber glass H-cell. For electrolysis, a potentiostat (Gamry Reference 3000, Gamry Instruments, USA) was used, while Ag-foam model electrodes, with a geometric surface area of 1 cm², functioned as the WE, a Pt mesh (Biologic) as the CE, and a Ag/AgCl electrode (Biologic) as the RE, respectively. Catholyte and anolyte compartments were separated by a Selemion anion exchange membrane (AGC Chemicals Europe, England). The electrolyte used for the CO_2_RR (aqueous 0.1 M KHCO_3_ solution) was prepared of powder (> 99.7% KHCO_3_, Carl Roth, Germany) and ultrapure water (Barnstead GenPure Pro, Thermo Scientific, USA), while the bottom of the catholyte chamber was continuously purged and saturated with CO_2_ gas (99.995 vol%, Air Liquide) with a flow rate of 20 mLmin^−1^, using a calibrated mass flow controller (Bronkhorst, Netherlands) to obtain a constant pH of 6.8. Chronoamperometric measurements lasted for 1 h, and different potentials were tested (−0.6 V to −1.3 V vs. RHE, iR corrected). The headspace of the custom-built H-cell was in-line connected to a gas chromatograph (GC) (GC-2014, Shimadzu, Japan) to detect and quantify the gaseous products of CO_2_RR. The product gases H_2_, CO, and CO_2_ were analysed after 1 h of electrolysis at the given potential and separated in HayeSep capillary columns connected in series (HayeSep Q + HayeSep R, 80/100 mesh, 2 + 2 m × 1/8 in.). The GC was equipped with a thermal conductivity detector (TCD) for the detection of H_2_. A flame ionization detector (FID) was used to detect CO (in the form of CH_4_ after passing through a methanation unit before the FID). Ar 5.0 was used as the carrier gas. The electrolyte was collected after 1 h of electrolysis at each potential and analysed for liquid products in a HPLC (LC2010, Shimadzu, Japan). The faradaic efficiencies (FE) of the specific products i were determined by dividing their partial currents by the total current using Eq. (1), where x_i_, z_i_, F, and ṅ are the volume fractions of the detected gaseous product, the number of electrons involved for a given reduction product, the Faraday constant, and the molar flow rate, respectively.

1 $${{{{{\rm{F}}}}}}{{{{{{\rm{E}}}}}}}_{{{{{{\rm{i}}}}}}}=\frac{{I}_{{{{{{\rm{i}}}}}}}}{{I}_{{{{{{\rm{t}}}}}}}}=\frac{{x}_{{{{{{\rm{i}}}}}}}\cdot {z}_{{{{{{\rm{i}}}}}}}\cdot F\cdot \dot{n}}{{I}_{{{{{{\rm{t}}}}}}}}$$

### Electrochemical surface area

Electrochemical surface areas (ECSA) were determined in a three-electrode set-up, using an in-house built H-cell and the same potentiostat as already used for the electrode preparation. All samples were measured in CO_2_-saturated electrolyte (0.1 M KHCO_3_, pH 6.8). Before recording the cyclic voltammograms (CVs) in the non-faradaic region (0.4 – 0.6 vs. RHE), the electrodes were subjected to a pre-conditioning step at the starting potential of 0.4 V vs. RHE for 15 s. The electrochemical double layer capacitance (C_DL_), used to calculate the ECSA from Eq. (3) was calculated by Eq. (2), with i as the average value of the charging current from the anodic and cathodic sweep, and ${{{{{\rm{v}}}}}}$, the scan rate, respectively. Ten different scan rates were applied (10–100 mVs^−1^) and the step size was set to 2 mV. The double layer capacitance of the reference (C_ref_ = 1.69 × 10^−5^ Fcm^−2^ ± 8 × 10^−8^ Fcm^−2^) that was found to be in good agreement with the literature^37^, was determined experimentally, using a mechanically polished Ag foil, as described in the section about electrode preparation from multiple CVs at ten different scan rates with the protocol described above.

2 $${C}_{{{{{{\rm{DL}}}}}}}=\frac{i}{v}$$

3 $${{{{{\rm{ECSA}}}}}}=\frac{{C}_{{{{{{\rm{DL}}}}}}}}{{C}_{{{{{{\rm{Ref}}}}}}}}\cdot {A}_{{{{{{\rm{geo}}}}}}}$$

### Gravimetric measurements

Mass differences (Δ-m) of the Ag catalysts before and after galvanostatic deposition were calculated with respect to the total masses of the foils measured with a Kern ABT 220-5DM precision balance and a resolution of 0.01 mg. The samples were therefore dried in laboratory atmosphere at room temperature for 24 h.

### Contact angle measurements

Contact angle measurements were conducted using an aqueous 0.1 M KHCO_3_ solution to simulate testing conditions during CO_2_RR in the H-cell set-up. Using the dispensing option of the SCA 20 software (DataPhysics Instruments, Germany), a drop of liquid (10 µl) was dispensed onto the centre of the tested electrode.

### Physisorption measurements

The gas physisorption measurements were conducted taking into account the IUPAC recommendations for gas physisorption^35^. Therefore, the measurements were performed with Krypton as adsorptive gas at 77 K. Krypton physisorption data were collected in the p/p_0_ range of 0.05–0.3 (fixed p_0_: 2.63 Torr) by using an Anton Paar QuantaTec ASiQ-MP-MP-AG set-up. The data were evaluated via Brunauer-Emmett-Teller (BET) method^38^ (either via multi-point or single-point fitting). Prior to the measurements the samples were cut in two pieces and were degassed at 120 °C for 12 h with subsequent testing of complete degassing.

### X-Ray diffraction

The Ag phase in the samples was analysed by X-ray diffraction (Philips X’Pert-MPD PW 3040/00) in Bragg-Brentano geometry using unfiltered Cu Kα radiation (PW3373/00 Cu LFF at 40 kV and 40 mA). The 10 mm by 20 mm samples were placed on a sample holder steel disc and the sample height was adjusted using a dial gauge (Käfer GmbH, Germany). Diffractograms were recorded with a proportional point detector (WP3011) at a step size of 0.02 ° 2θ and a scan rate of 0.12° 2θ/min between 10° and 90° 2θ. Cu K_α2_ reflections were removed post measurement with Highscore software. Instrumental broadening *β*_inst_ was calculated from the diffractogram of a Sapphire single crystal. The full width at half maximum (FWHM) of the reflections were plotted over the 2θ angle and linearly fitted. The fit function allowed to determine the instrumental broadening at a given 2θ angle.

All peaks were fitted using the split pseudo-Voigt function using Profex 5^39^ and the widths of the Ag reflections were calculated from the half width at half maximum (HWHM) by

4 $$\beta ={{{{{\rm{HWHM}}}}}}\sqrt{\frac{{{{{{\rm{\pi }}}}}}}{{{{{{\rm{ln}}}}}}(2)}}$$

For the determination of the crystallite sizes as well as strain of the Ag samples the instrumental broadening was subtracted assuming Gaussian convolution:

5 $${\beta }_{{{{{{\rm{hkl}}}}}}}^{2}={\beta }_{{{{{{\rm{measured}}}}}}}^{2}+{\beta }_{{{{{{\rm{instrumental}}}}}}}^{2}$$

Crystallite sizes were calculated from the (111) reflection using the Scherrer equation:

6 $$L=\frac{{{{{{\bf{K}}}}}}\lambda }{\beta \;{{\cos }}{{\theta }}}$$

Micro strain was determined by applying the Williamson-Hall method. The Williamson-Hall equation is derived by the deconvolution of two Lorentzians of the breadths for inner crystallite strain and crystallite size:^40^

7 $$\beta \;{{\cos }}{{\theta }}=C\varepsilon \;{{\sin }}{{\theta }}+\frac{{{{{{\bf{K}}}}}}\lambda }{L}$$

where beta is the breadth of the measured reflection, θ is the Bragg angle, *C* is a constant of usually 4 to 5, ε is the dimensionless inhomogeneous strain, **K** is a constant according for the shape of the crystallites, where 0.9 is usually a suitable estimation, *λ* is the wavelength of the incident X-ray beam (*λ* (Cu Kα1) = 15.4056 nm) and *L* is the crystallite size. Plotting sin θ versus *β* cos θ gives a linear relation, in which the strain is then obtained from the slope.

### Scanning electron microscopy

High resolution images were recorded using the Secondary Electron Detector (SE) of a scanning electron microscope (Zeiss Ultra plus, Carl Zeiss, Germany). To be able to investigate a representative number of macropores for each tested sample, 16 pictures were stitched together using the software package Fiji (ImageJ 1.52p)^41,42^. Consequently, an area of ~15.29 mm^2^ could be analysed simultaneously.

### Laser confocal scanning microscopy

Topographic investigation was conducted using a LCSM (900 M, Carl Zeiss, Germany), equipped with a laser source with a wavelength of 405 nm and a 50× lens. Before each measurement, the sample was placed centrically to the laser source and an area of 1150 µm × 1150 µm was scanned. The results were processed and analysed using the ConfoMap ST Software (Carl Zeiss, Germany). All images were levelled using a linear function before height profiles and histograms were extracted. The volumes of the Ag bulk, the Ag foams and the macro porosity were calculated by the following equations:

8 $${V}_{{{{{{\rm{total}}}}}}}={x}_{{{{{{\rm{total}}}}}}}\cdot {y}_{{{{{{\rm{total}}}}}}}\cdot {z}_{{{{{{\rm{total}}}}}}}$$

9 $${V}_{{{{{{\rm{Ag}}}}}}}={\int }^{{{{{{{\rm{y}}}}}}}_{{{\max }}}}_{{{{\!\!{{{{\rm{y}}}}}}}_{{{\min }}}}}{\int }^{{{{{{{\rm{x}}}}}}}_{{{\max }}}}_{{{{\!\!{{{{\rm{x}}}}}}}_{{{\min }}}}}z\cdot {{{{{\rm{d}}}}}}x\cdot {{{{{\rm{d}}}}}}y$$

10 $$\varPhi =\frac{{V}_{{{{{{\rm{Ag}}}}}}}}{{V}_{{{{{{\rm{total}}}}}}}}$$

### Focused ion beam—scanning electron microscopy

To analyse the nanostructure of the Ag foam electrodes in detail in 2D, all electrodes have been measured by SEM and FIB technology, using a Zeiss Crossbeam 340 Gallium-FIB/SEM. To analyse the sample, a 10 mm × 10 mm piece was mechanically cut from the middle of the electrode and horizontally mounted on a sample holder, using a carbon pad for fixing. For the coarse cutting step an energy of 30 keV and a current of 15 nA has been applied. The polishing step has been carried out using 30 keV and 7 nA. The SEM image was taken using an acceleration voltage of 1 keV and a pixel size of 5 nm.

To analyse the foam structure in 3D, the samples were measured by FIB tomography. For this purpose, a piece of 5 mm × 10 mm was mechanically cut from the middle of the electrode and embedded in a slowly hardening resin (Buehler, EpoThin^TM^ 2, Epoxy Resin and Hardener). Subsequently, the sample was grinded vertically to the electrode’s plane to free the cross section of the electrode. Afterwards, the sample was mounted on a sample holder, using silver and copper tape. For the coarse cutting step an energy of 30 keV and a current of 7 nA were used. The tomographies were carried out using 30 keV and 3 nA at an acceleration voltage of 1 keV and a pixel size of 10 nm. For each sample, a minimum volume of at least 16 × 11 × 6 µm³ has been reconstructed. The reconstruction and segmentation of the cut volume as well as further calculations to analyse the nano porous foam structure have been performed by Fiji (ImageJ 1.52p)^41,42^.

## Supplementary information

Supplementary Information

## Data Availability

The data that support the findings of this study are available from the corresponding author upon reasonable request.
