# Supplementary material for: Multi-scale morphology characterization of hierarchically porous silver foam electrodes for electrochemical CO2 reduction
Source: Commun Chem. 2023 Mar 16;6:50. doi: 10.1038/s42004-023-00847-z (PMC10020469; doi:10.1038/s42004-023-00847-z)
Supplement: Supplementary file 1 — Supplementary Information [file 42004_2023_847_MOESM1_ESM.pdf]

Supplementary Information

Multi-scale Morphology Characterization of Hierarchically Porous Silver Foam Electrodes for Electrochemical CO_2 Reduction

Hendrik Hoffmann[1*], Melanie Cornelia Paulisch-Rinke[2], Marius Gernhard[1], Yannick Jännsch[3], Jana Timm[4], Carola Brandmeir[1], Steffen Lechner[1], Roland Marschall[4], Ralf Moos[3], Ingo Manke[2], and Christina Roth[1]

CO_2RR results after 2 h of potentiostatic operation at elevated potential

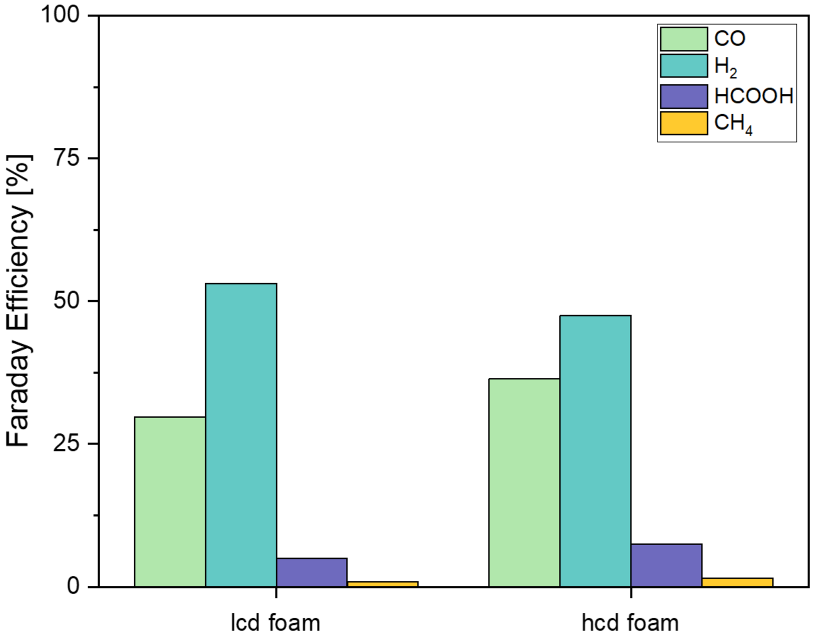

Figure S-1: Faradaic efficiencies of the evaluated products of lcd and hcd foam electrodes (deposited at 0.5 Acm^{-2} 80 s and 2.0 Acm^{-2} 20 s) after 2 h of potentiostatic CO_2RR operation at -1.3 V vs. RHE.

Scanning electron microscopy stitched 4515.2 µm ×3386.4 µm sized images

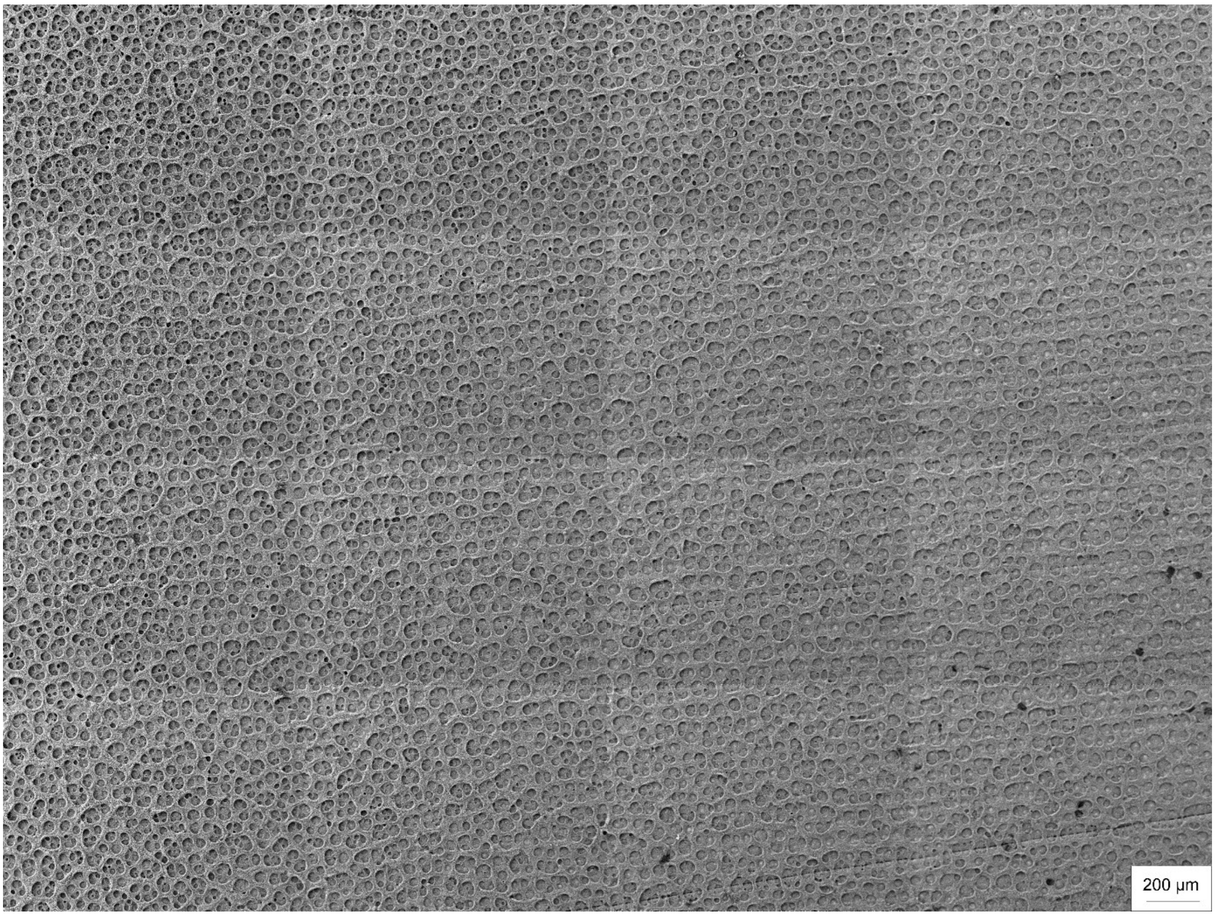

Figure S-2: 2.0 Acm-2 @ 10 s.

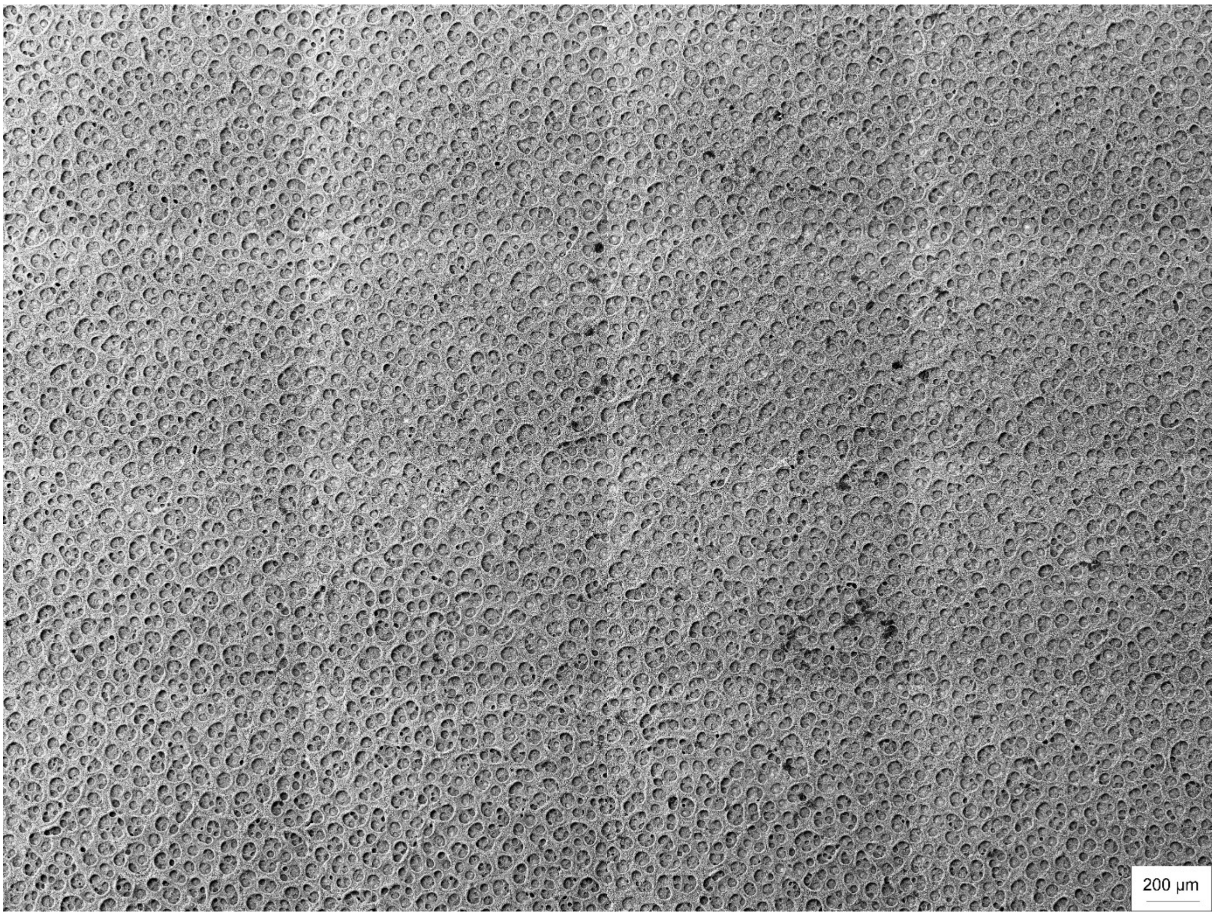

Figure S-3: 2.0 Acm-2 @ 15 s.

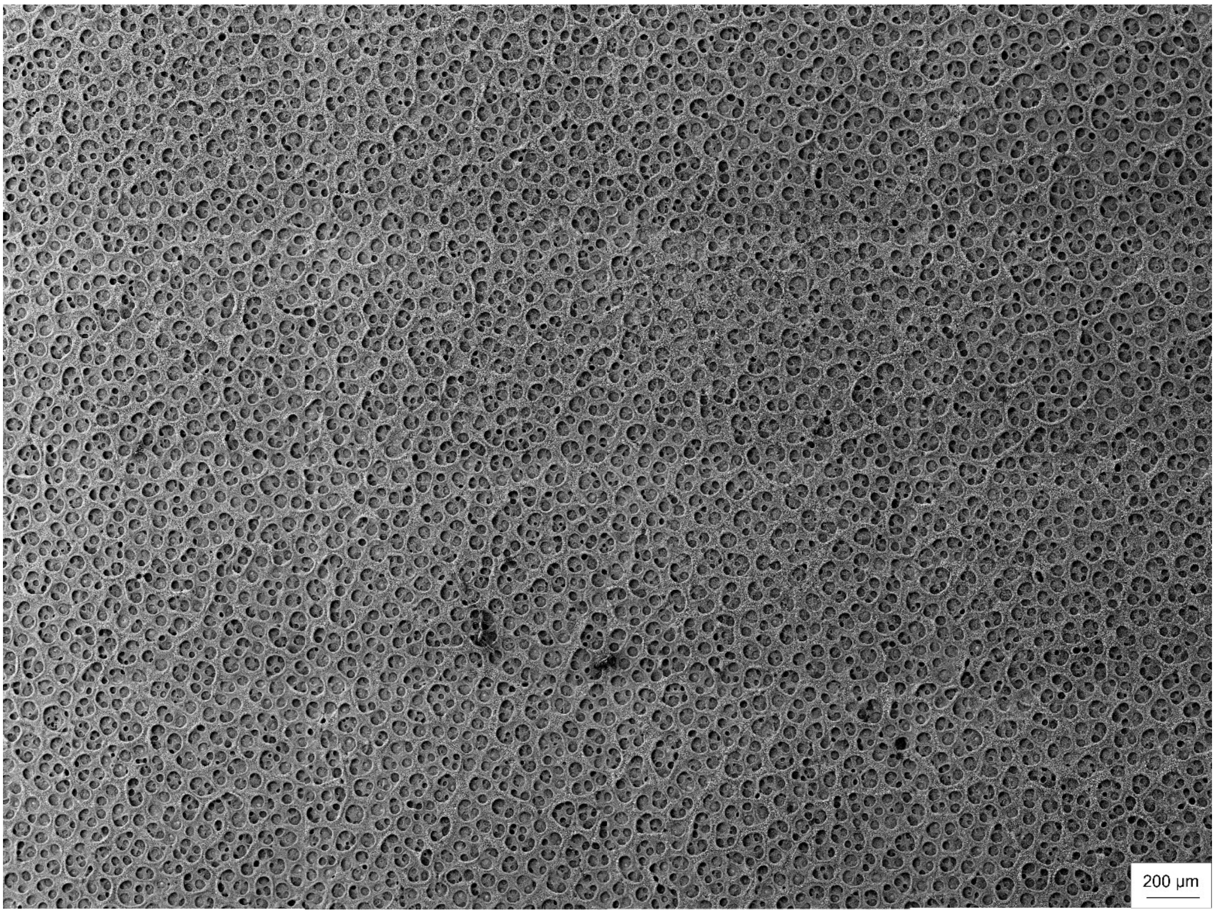

Figure S-4: 2.0 Acm-2 @ 20 s.

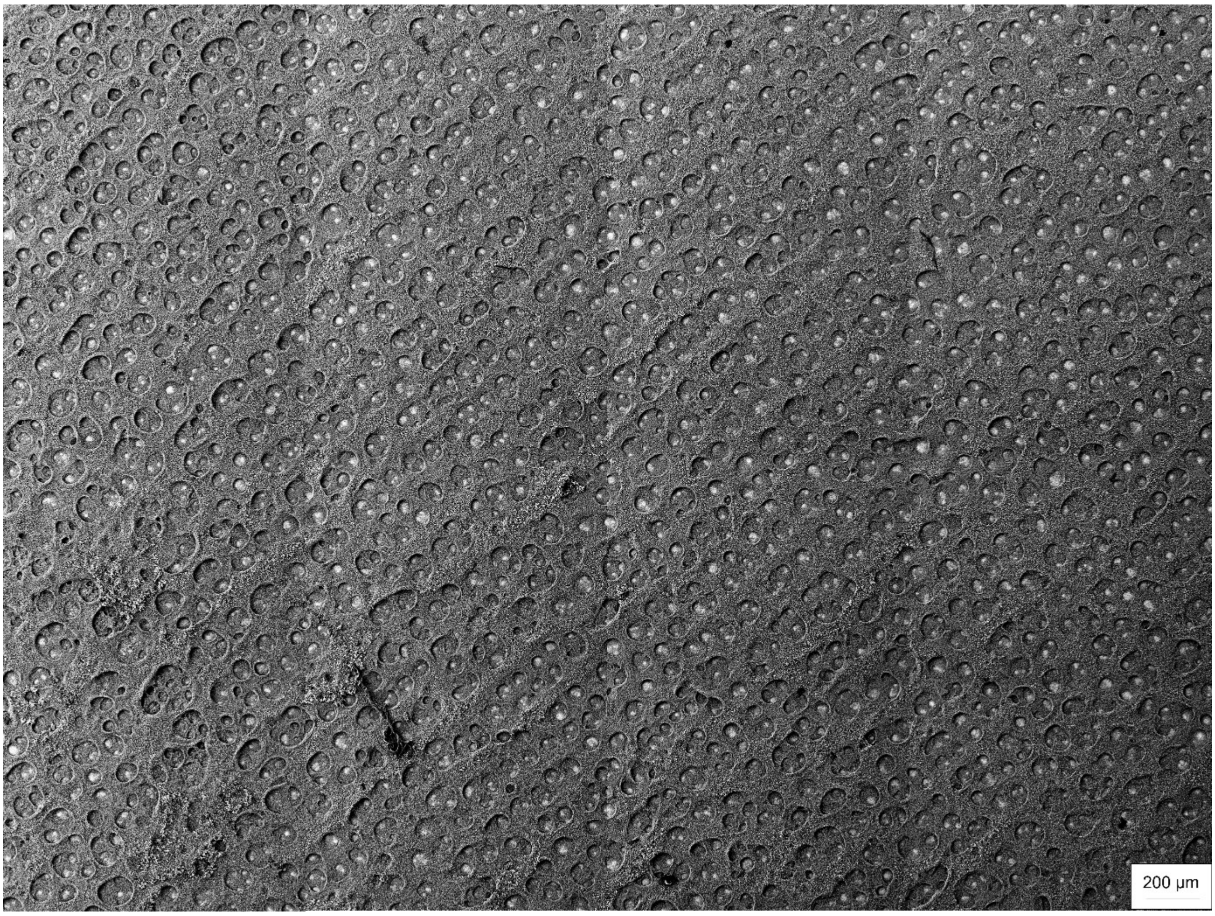

Figure S-5 0.5 Acm-2 @ 40 s.

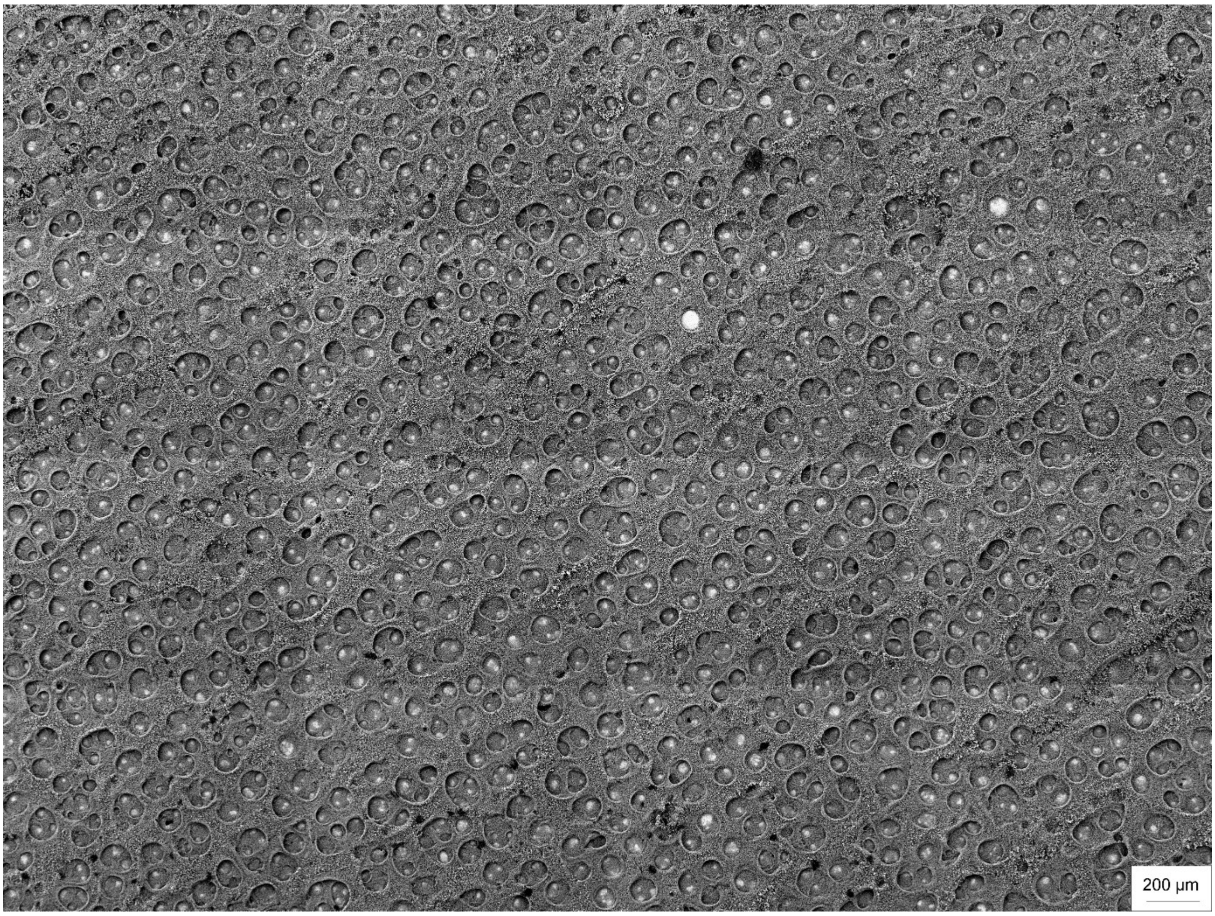

Figure S-6: 0.5 Acm-2 @ 60 s.

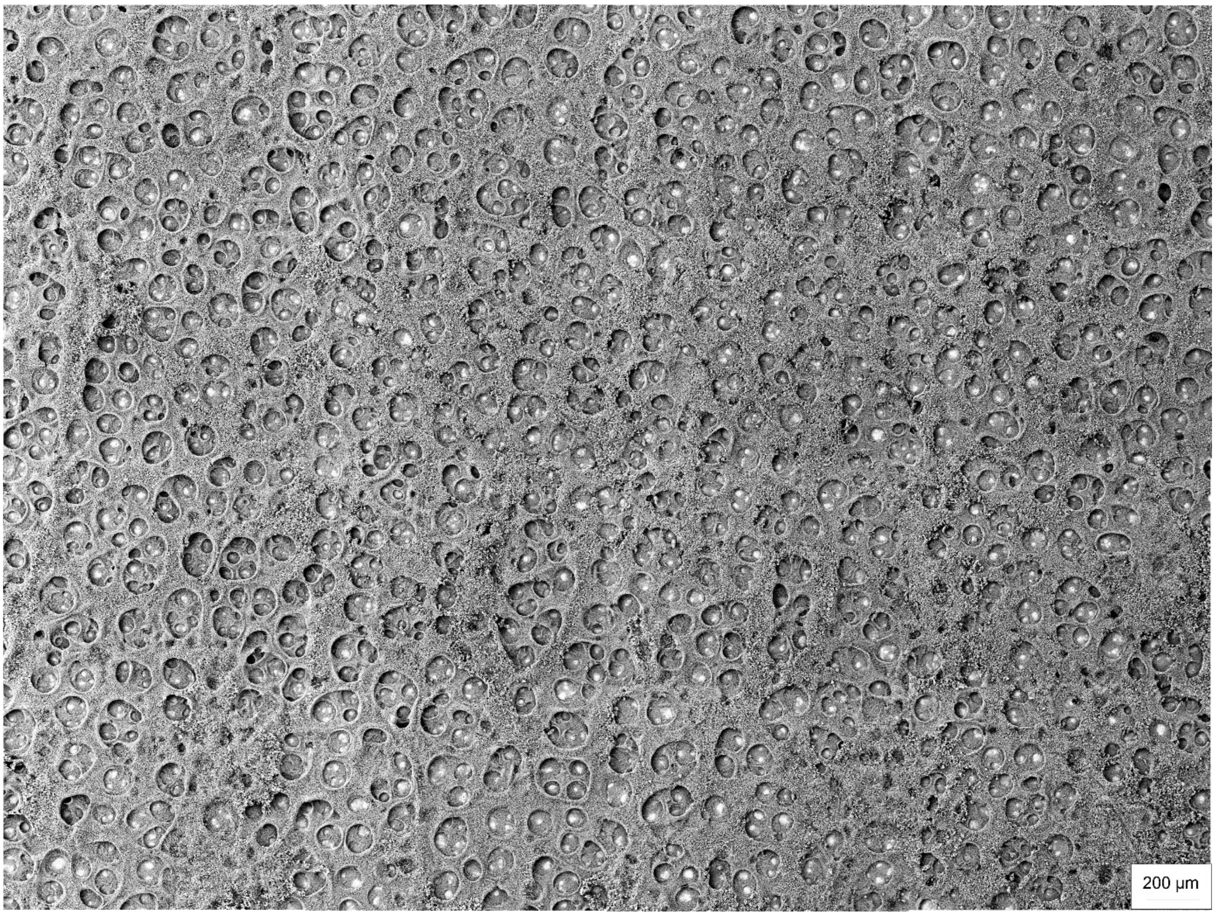

Figure S-7: 0.5 Acm-2 @ 80 s.

Scanning electron microscopy images of Ag foams before and after CO_2RR

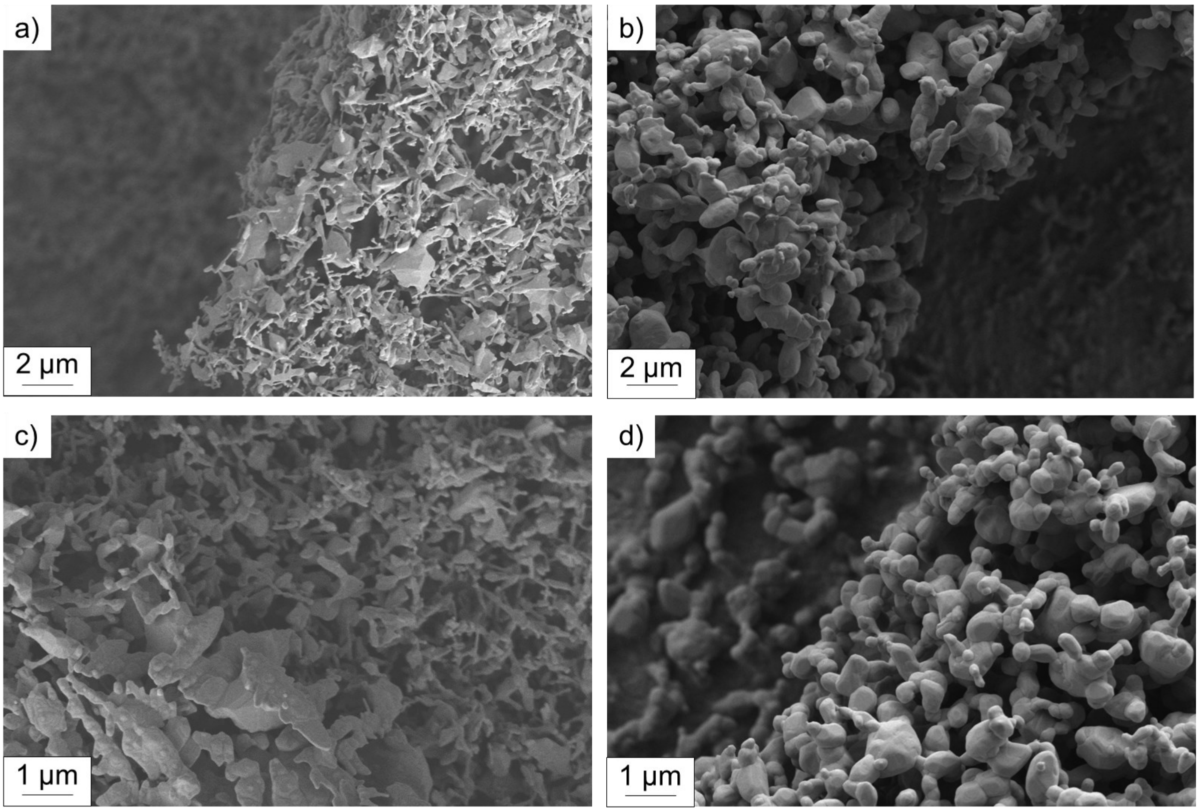

Figure S-8: SEM images of foam electrodes in pre and post CO_2RR conditions, tested at -1.3 V vs. RHE for lcd foams (a) and (c) and hcd foams (b) and (d). After operation, a „smoothening“ of the plate-like rough surfaces is observed.

Reconstruction of laser confocal scanning microscopy images

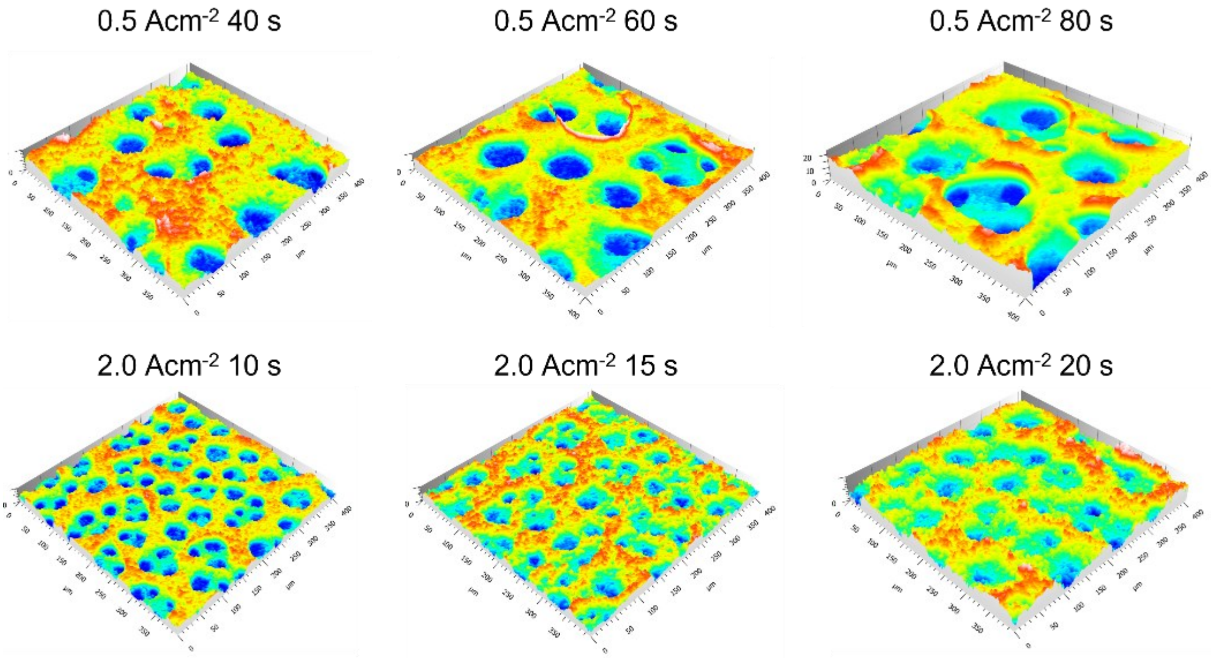

Figure S-9: 3D reconstruction of Ag foam electrodes deposited with 0.5 Acm^{-2} (upper row) and 2.0 Acm^{-2} (lower row).

Electrochemical Performance of hcd and lcd foams in CO_2RR

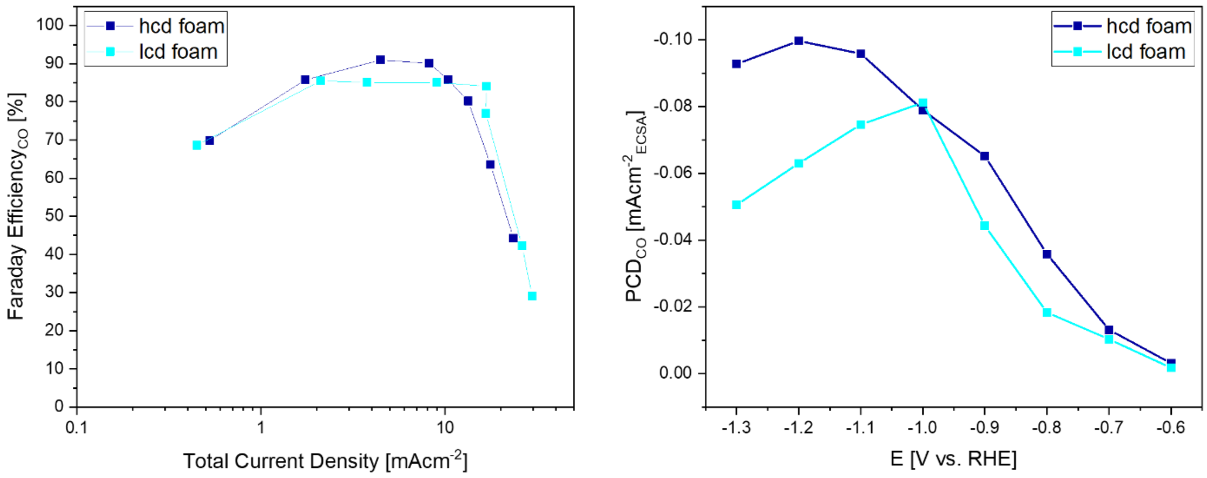

Figure S-10: FE_{CO} as a function of total geometric current density applied during CO_2RR (left) and partial current density for CO formation as a function of applied electrode potential during potentiostatic CO_2RR operation (right).

Graphical interpretation of structure-performance relationship

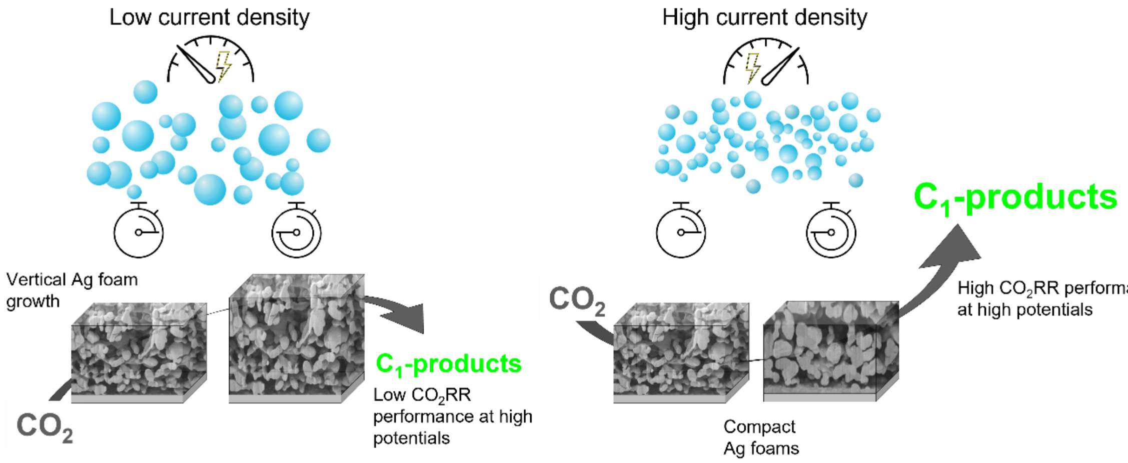

Figure S-11: Graphical representation of the influence of deposition conditions at low and high current densities to produce Ag foams, leading to higher stability and performance during CO_2RR with respect to the production of C_1-products.
